# Retraction: Study on the deterioration characteristics of aeolian sand concrete under the coupling effect of multiple factors in harsh environments

**DOI:** 10.1371/journal.pone.0346394

**Published:** 2026-04-06

**Authors:** The PLOS One Editors

The *PLOS One* Editors retract this article [1] due to concerns about peer review and compliance with PLOS policy on Manipulation of the Publication Process.

GL and BG did not agree with the retraction. CZ, HH, and HF either did not respond directly or could not be reached.
